# Confining He Atoms in Diverse Ice-Phases: Examining the Stability of He Hydrate Crystals through DFT Approaches

**DOI:** 10.3390/molecules28237893

**Published:** 2023-12-01

**Authors:** Raquel Yanes-Rodríguez, Rita Prosmiti

**Affiliations:** Institute of Fundamental Physics (IFF-CSIC), CSIC, Serrano 123, 28006 Madrid, Spain; raquelyr@iff.csic.es

**Keywords:** electronic structure calculations, DFT approaches, molecular interactions, gas hydrates, crystal He-filled ice phases

## Abstract

In the realm of solid water hydrostructures, helium atoms have a tendency to occupy the interstitial spaces formed within the crystal lattice of ice structures. The primary objective of this study is to examine the stability of various ice crystals when influenced by the presence of He atoms. Presenting a first attempt at a detailed computational description of the whole energy components (guest–water, water–water, guest–guest) in the complete crystal unit cells contributes to enhancing the knowledge available about these relatively unexplored helium–water systems, which could potentially benefit future experiments. For this purpose, two different ice structures were considered: the previously established He@ice II system, and the predicted (but currently nonexistent) He@ice XVII system. One of the main features of these He-filled structures is the stability conferred by the weak van der Waals dispersion forces that occur between the host lattice and the guest atoms, in addition to the hydrogen bonds established among the water molecules. Hence, it is crucial to accurately describe these interactions. Therefore, the first part of this research is devoted examining the performance and accuracy of various semi-local and non-local DFT/DFT-D functionals, in comparison with previous experimental and/or high-level computational data. Once the best-performing DFT functional has been identified, the stability of these empty and He-filled structures, including different number of He atoms within the lattices, is analysed in terms of their structural (lattice deformation), mechanical (pressure compression effects) and energetic properties (binding and saturation energies). In this manner, the potential formation of these structures under zero temperature and pressure conditions can be evaluated, while their maximum storage capacity is also determined. The obtained results reveal that, despite the weak underlying interactions, the He encapsulation has a rather notable effect on both lattice parameters and energetics, and therefore, the guest–host interactions are far from being negligible. Besides, both ice crystals are predicted to remain stable when filled with He atoms, with ice XVII exhibiting a higher capacity for accommodating a larger number of guest atoms within its interstitial spaces.

## 1. Introduction

Due to their small size and weak interaction with water molecules, helium atoms tend to occupy interstitial spaces within the crystal lattice of ice-like structures, such as hexagonal ice (I${}_{h}$) or ice II, rather than forming stable clathrate-like structures [1,2,3,4]. Nonetheless, the information available about these He-filled structures is quite limited, with the helium–water system remaining relatively unexplored. Arnold et al. made a prediction in 1971 that helium could potentially enter the open channels of both ice I${}_{h}$ and ice II [5]. Prior to this, evidence for helium in ice I${}_{h}$ had already been discovered [6,7], and later solubility studies provided further confirmation [8,9,10]. It was not until 1988 that the first experimental evidence for the formation of helium inclusion compounds in ice II at pressures exceeding 0.28 GPa was presented by Londono et al. [1]. A few years later, a more detailed neutron diffraction experiment on this structure was conducted [2]. Lobban et al. expanded on the discussion of He-filled ice II by comparing it with refined neutron diffraction data for pure ice II [11]. In 2003, neutron-diffraction measurements on He-affected ice I${}_{h}$ samples were reported by Wang and Li [12]. New experimental data on He-filled ice I${}_{h}$ up to 120 bar were published in 2013 [13]. It was necessary to await until 2018 for the publication of novel data concerning the first experimental confirmation of helium inclusion in sII clathrate hydrate (ice XVI) and ice I${}_{h}$. In the meantime, a few theoretical studies on pure He-filled ices [14,15,16,17,18], as well as computational and experimental findings in helium containing mixtures, have been published [19,20,21,22].

Despite the seemingly comprehensive research on He-filled ice crystals, there is actually a scarcity of valuable data on He@hydrate interactions. This deficiency motivates us to further expand the current knowledge available about such ice crystals in the presence of He atoms by investigating their stability through DFT computations. The selection of the He atom as a guest is driven not just by the limited research on helium hydrates, but also by its potential as a promising candidate for generating novel ice structures. The unique nature of He allows it to form interactions that can sufficiently stabilize certain inclusion compounds, and yet be weak enough to facilitate its removal from the host structure, thus enabling the stabilization/formation of new structures or the incorporation of alternative guest molecules [23]. This advantageous feature has already been harnessed in a Ne hydrate, where the Ne atoms initially stabilized the sII clathrate hydrate and were subsequently extracted, resulting in the formation of ice XVI [24].

Within this context, computational investigations emerge as advantageous tools that hold the potential to guide and accelerate the discovery of novel materials and reactions [25,26,27,28,29]. The availability of high-performance computing platforms and supercomputers empowers researchers to address computationally intensive tasks, such as molecular dynamics simulations, electronic structure calculations, and chemical reaction modeling [30,31,32,33]. Moreover, the development and implementation of diverse quantum chemistry methodologies, such as density functional theory (DFT), *ab initio* electronic structure methods, and machine learning approaches, have expanded the scope of chemical systems that can be studied with unprecedented precision and efficiency [34,35,36,37]. Thus, a wide range of properties can be explored, offering insights into the structure, energetics, spectroscopy or reactivity of molecules, materials and reactions.

Hence, building upon our previous endeavours in studying the stability of helium clathrate hydrates [38,39,40], our current research focuses on examining the impact of confining He atoms within the channels of ice crystals through DFT computations. To this end, two different types of ices were selected: ice II, which has already been experimentally observed in the presence of He atoms [2], and a recently discovered form of ice, known as ice XVII [41], which has been successfully filled with Ne atoms in the laboratory [42]. This novel structure has garnered the attention of numerous researchers [43,44,45,46,47], and since there is no absolute understanding of its stability in the presence of helium atoms, we have opted to include it in our study. As far as we are aware, this is the first time that theoretical data on its stability will be provided. Hence, this study is a first attempt to describe, through first-principles computations, the interactions (guest–guest, guest–host, host–host) that govern the stability of these ice crystals in the presence of He atoms in terms of structural, mechanical, and energetic properties. Furthermore, we will also delve into the effects of multiple occupancy, aiming to determine the maximum storage capacity of these systems.

## 2. Results and Discussion

This investigation is divided into two distinct parts. The first part involves a benchmark study on the crystal structures of ice II and ice XVII. The objective is to evaluate the performance of different DFT-D functionals that have been recognized as effective in describing ices and clathrate hydrates in the literature [38,45,48,49,50,51,52,53,54]. In addition, the benchmark study encompasses ice II and ice XVII structures containing one or multiple He atoms, respectively, alongside their respective empty ice II/ice XVII lattices, in order to consider the impact of an increasing number of He atoms occupying the interstitial spaces within the ice lattices. Once the most suitable DFT-D functional for accurately describing the interactions in ices has been determined, the second part of the investigation focuses on analyzing the stability of ice II and ice XVII frameworks. To identify the most favorable configuration, several He-fillings are considered, each involving a different number of He atoms that occupy the voids.

### 2.1. Simulation Cells, Computational Details and Lattice Energies

**Figure 1:**
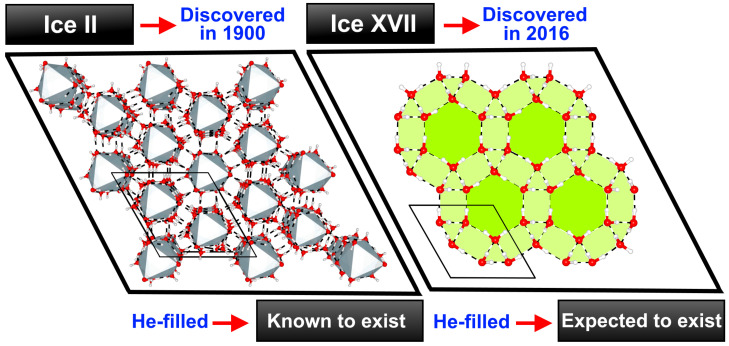
View along *c*-axis of ice II and ice XVII unit cells and corresponding extended crystal systems (plotted using VESTA [56]), with oxygen, hydrogen atoms in red and white color, respectively, while hydrogen bonds are shown as black lines.

In Figure 1 the c-axis view of the two structures under study is depicted. Ice II is a proton-ordered form of ice, composed of two different types of hexagonal water rings, one chair-form and one almost flat, which are alternatively stacked along the *c*-axis, forming columns with interstitial spaces between rings [55]. The hexagonal unit cell (R$\bar{3}$ space group), with lattice parameters a = b ≠ c and angles $\alpha =\beta =90^{\circ }$ and $\gamma =120^{\circ }$, is formed by 36 water molecules. The structure of ice XVII is characterized by hexagonal helices that spiral along the c-axis. These helices are constructed from sheets that consist of hydrogen-bonded pentagonal water rings. The hexagonal unit cell (P6${}_{1}$22 space group) is formed by a water pentamer hydrogen-bonded to a water molecule. Their structures were determined by neutron diffraction experiments and reported in refs. [2,43], respectively. Various configurations containing a varying number of He atoms were also considered. One, two and three He atoms are confined within the voids of ice II, identified as He${}_{\left(1\right)}$@ice II (H${}_{2}$O:He ratio, called hydration number, 36:6), He${}_{\left(2\right)}$@ice II (H${}_{2}$O:He, 36:12) and He${}_{\left(3\right)}$@ice II (H${}_{2}$O:He, 36:18), respectively. Since the ice XVII channels have a zig-zag form, we will distinguish between two types (right/left) of interstitial spaces. Thus, He${}_{\left(1/1\right)}$@ice XVII (H${}_{2}$O:He, 6:2), He${}_{\left(1/2\right)}$@ice XVII (H${}_{2}$O:He, 6:3), He${}_{\left(2/2\right)}$@ice XVII (H${}_{2}$O:He, 6:4) and He${}_{\left(3/3\right)}$@ice XVII (H${}_{2}$O:He, 6:6) are contemplated.

Electronic structure DFT calculations were performed for both empty and He-filled ice II and ice XVII structures using the QE code [57,58,59]. A benchmark study is first carried out including the semi-local exchange–correlation PW86PBE [60,61] and revPBE [62] GGA (generalized gradient approximation) functionals, along with the XDM [63,64] and D3(0) [65] dispersion schemes as implemented in QE, and the D4 correction as a post-processing tool through the DFT-D4 program [66,67,68,69], as well as the non-local van der Waals-inclusive optB88-vdW [70], vdW-DF [71] and vdW-DF2 [72] functionals, with the dispersion contribution as an integral part of the exchange-correlation interaction. In this way, we investigate few selected functionals with different exchange-correlation expressions, as well as dispersion-correction schemes. The plane-wave pseudopotential approach within the projector-augmented-waves (PAW) method [73] was employed using the standard PBE-based pseudopotentials supplied within QE. Moreover, several single-point runs were carried out to check the convergence of the energy cutoff for the plane-wave expansion of the wave functions, Ecut_wfc, and for the charge density, Ecut_rho, resulting in a selection of 360/440 Ry (4898/5986 eV) for Ecut_wfc and 90/100 Ry (1224/1360 eV) for the Ecut_rho for the empty and He-filled ice II/ice XVII structures, respectively. A Monkhorst-Pack 2 × 2 × 2/6 × 6 × 6 k-point grid [74] in the reciprocal space was used per unit cell in the ice II/ice XVII systems, while calculations for the isolated He and H${}_{2}$O molecules were performed at the Γ-point in a cubic simulation cell of volume 30 × 30 × 30 Å, considering the Makov–Payne method [75] of electrostatic interaction correction for these aperiodic systems. As for the geometry optimizations, all atomic positions were relaxed using the the Broyden–Fletcher–Goldfarb–Shanno (BFGS) quasi-newton algorithm with convergence criterion for the components of energy and forces being smaller than 10${}^{-4}$ Ry (10${}^{-3}$ eV) and 10${}^{-3}$ Ry/bohr (10${}^{-2}$ eV/bohr), respectively.

The cohesive energies per water molecule for the He-filled ices were calculated as:

(1) $$\Delta E_{coh}^{He_{N}@ice}=\frac{E_{opt}^{He_{N}@ice}\left(a;c\right)-M\cdot E^{H_{2}O}-N\cdot E^{He}}{M},$$

where E${}_{\mathrm{opt}}^{{\mathrm{He}}_{N}@\mathrm{ice}}\left(a,c\right)$ is the total energy of the occupied He${}_{N}$@ice unit cell obtained by structural relaxation at each lattice constants a and c, E${}^{H_{2}O}$ and E${}^{\mathrm{He}}$ are the total energies of the isolated ground-state H${}_{2}$O molecules and He atoms, respectively, M is the total number of water molecules (M = 36/6 for ice II/ice XVII) and N corresponds to the total number of He atoms, which in this case corresponds to N = 6/12/18 for the He${}_{\left(1\right)}$@ice II, He${}_{\left(2\right)}$@ice II and He${}_{\left(3\right)}$@ice II and N = 2/3/4/6 for the He${}_{\left(1/1\right)}$@ice XVII, He${}_{\left(1/2\right)}$@ice XVII, He${}_{\left(2/2\right)}$@ice XVII and He${}_{\left(3/3\right)}$@ice XVII configurations, respectively.

Regarding the empty ice crystals, the cohesive energy is equivalently computed as:

(2) $$\Delta E_{coh}^{ice}=\frac{E_{opt}^{ice}\left(a^{\prime },c^{\prime }\right)-M\cdot E^{H_{2}O}}{M},$$

where E${}_{\mathrm{opt}}^{\mathrm{ice}}\left(a^{\prime },c^{\prime }\right)$ stands for the optimized total energy of the empty ice unit cells at the different values of lattice constants $a^{\prime }$ and $c^{\prime }$. Another energetic term used to analyse the stability of these compounds is the binding energy resulting from the encapsulation of the He atoms in the empty ice systems, which is given by

(3) $$\Delta E^{He_{N}@ice}=E_{opt}^{He_{N}@ice}\left(a_{0},c_{0}\right)-E^{ice}\left(a_{0},c_{0}\right)-N\cdot E^{He}$$

where $E_{\mathrm{opt}}^{{\mathrm{He}}_{N}@\mathrm{ice}}\left(a_{0},c_{0}\right)$ corresponds to the total energy of the occupied He${}_{N}$@ice structures obtained via structural relaxation at the equilibrium lattice constants a${}_{0}$ and c${}_{0}$, and E${}^{\mathrm{ice}}\left(a_{0},c_{0}\right)$ is the total energy of the empty crystals at the equilibrium lattice constants a${}_{0}$ and c${}_{0}$. This quantity is also given in a per He atom basis. Finally, the energy corresponding to the saturation of all the channels in the ices, at a${}_{0}$ and c${}_{0}$, is calculated as:

(4) $$\Delta E_{sat}=E_{opt}^{He_{N}@ice}\left(a_{0},c_{0}\right)-E_{opt}^{crystal}\left(a_{0},c_{0}\right)-E_{opt}^{N\cdot He}\left(a_{0},c_{0}\right),$$

where $E_{\mathrm{opt}}^{N\cdot \mathrm{He}}\left(a_{0},c_{0}\right)$ is the total energy of the entire number of He atoms obtained by structural relaxation at the equilibrium lattice constants, a${}_{0}$ and c${}_{0}$.

### 2.2. Benchmarking DFT-D Calculations

The semi-local revPBE and PW86PBE functionals were examined, along with the optB88-vdW, vdW-DF and vdW-DF2 functionals in order to investigate the impact of non-local dispersion effects. Dispersion corrections, such as those obtained from the XDM, D3(0) and D4 schemes [63,64,65,67,68,69], were also considered. For this purpose, geometric optimizations of all atomic positions of both He-filled and empty crystal structures of ice II and ice XVII were computed. In the case of ice II, the structure is filled with only one 1 He per interstitial space He${}_{\left(1\right)}$@ice II, while ice XVII contains 3 He atoms per void He${}_{\left(3/3\right)}$@ice XVII. In this manner, we can assess the effectiveness of the DFT functionals under three distinct conditions: empty structures (host–host interactions), structures filled with a single He atom per interstice (guest–host interactions), and structures filled with multiple He atoms (guest-guest interactions).

Full geometry optimizations were performed through DFT/DFT-D computations on the empty and He-filled ice structures at various lattice parameters, a, while keeping the crystal atomic radius between the c and a lattice parameters (r = c/a) constant. Before carrying out the whole benchmark study, total energies at a wide range of r and a values were calculated employing only the PW86PBE/-XDM/-D4 functional (given its lower computational cost), so as to identify the most energetically favourable r curve for both the empty and He-filled ice II and ice XVII systems. Specifically, the values of r and a for ice II/He${}_{\left(1\right)}$@ice II and ice XVII/He${}_{\left(3/3\right)}$@ice XVII were chosen to be within the ranges of 0.46–0.50 and 12.0–14.6 Å, and 0.94–0.98 and 5.8–7.4 Å, respectively. Figure S1 in the Supplementary Material shows the cohesive energies per water molecule plotted against a for different r values, with r = 0.48 and r = 0.96 yielding the lower energy curves for the empty/He-filled ice II and ice XVII structures, respectively, despite the difference between the curves at various r values being insignificant.

**Figure 2:**
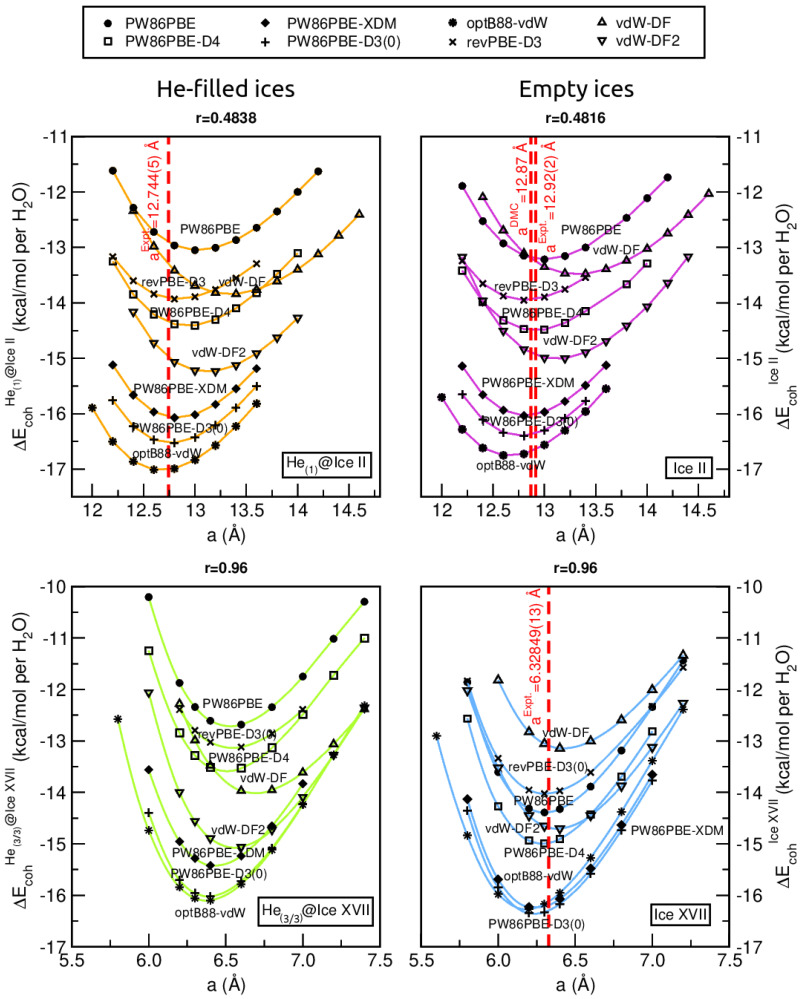
Cohesive energies, in kcal/mol per water molecule, of the He${}_{\left(1\right)}$@ice II (**upper panels**) and He${}_{\left(3/3\right)}$@ice XVII (**lower panels**) crystalline structures, together with their empty analogs, as a function of the lattice constant a and crystal atomic radius r values. Solid lines corresponds to the MEOS fits, while the values of experimental/theoretical equilibrium lattice constants are drawn with vertical red dashed lines. See Refs. [2,76] for experimental values extrapolating at zero T-P conditions for the He@ice II and ice II, respectively, and Ref. [76] for the DMC value of ice II. The experimental value for ice XVII at T = 25 K is taken from Ref. [43].

Therefore, hereafter, we focus the benchmark analysis on r = 0.4816/0.4838 (readjusted on the basis of experimental values [2,43]) for ice II/He${}_{\left(1\right)}$@ice II, and r = 0.96 for both ice XVII and He${}_{\left(3/3\right)}$@ice XVII. Figure 2 displays the cohesive energies per water molecule computed with the whole set of semi-local and non-local DFT functionals for both He-filled and empty ice structures. The DMC and/or experimental values available in the literature [2,43,76] are used as a guideline for elucidating the best-performed functional. Two characteristics are noteworthy from the analysis of these plots. On the one hand, it is observed that the vdW-DF and vdW-DF2 functionals cause the curves to shift towards larger a values, as expected due to their ability to account for long-range van der Waals interactions, which results in an increase in the equilibrium distance between particles. This effect is pronounced in He-filled and empty ice II, as well as in He${}_{\left(3/3\right)}$@ice XVII. On the other hand, although the inclusion of He atoms barely has any effect on the lattice of ice II, their presence in ice XVII has a significant impact. Thus, a displacement of the curve to larger a values is observed, indicating lattice expansion. This expansion suggests that the six He atoms within the interstitial spaces in the channel exert a repulsive effect.

In turn, with the aim of obtaining the equilibrium lattice constants, $a_{0},c_{0}$ (or $a_{0}^{\prime },c_{0}^{\prime }$), and the corresponding equilibrium cohesive energies, the calculated total energies for the He-filled and empty ices as a function of the lattice constant a and crystal atomic radius r are fitted to the Murnaghan’s equation of state (MEOS) [77] (see solid lines in Figure 2), which represents a relation between the volume and the energy, $E\left(V\right)=E_{0}+B_{0}\;V_{0}[\frac{1}{B_{0}^{\prime }\left(B_{0}^{\prime }-1\right)}{\left(\frac{V}{V_{0}}\right)}^{1-B_{0}^{\prime }}$$+\frac{1}{B_{0}^{\prime }}\frac{V}{V_{0}}-\frac{1}{B_{0}^{\prime }-1}]$, where B${}_{0}$ and B${}_{0}^{\prime }$ are the modulus of incompressibility and its first derivative with respect to the pressure, respectively.

The resulting equilibrium lattice parameters, as well as equilibrium energies and bulk moduli (B${}_{0}$ and B${}_{0}^{\prime }$ at zero pressure) are listed in Table S1 in the Supplementary Material. As discussed above, the vdW-DF and vdW-DF2 overestimate the $a_{0}$ constants, yielding values ∼0.5 and 0.3 Å larger than the ${\mathrm{He}}_{\left(1\right)}$@ice II/ice II experimental ones (12.74/12.92 Å) [2,76], respectively, and ∼0.1 Å larger than the ice XVII experimental one (6.33 Å) [43]. In fact, among the methods evaluated, vdW-DF followed by vdW-DF2 and optB88-vdW show the highest % error. In contrast, revPBE-D3(0) exhibits the smallest average % error (0.6%), deviating from the experimental values by only 0.1 Å for ${\mathrm{He}}_{\left(1\right)}$@ice II/ice II and 0.03 Å for ice XVII. The PW86PBE, PW86PBE-D4 and PW86PBE-D3(0) functionals proportionate to the closest values for ice XVII, ice II and ${\mathrm{He}}_{\left(1\right)}$@ice II, respectively.

**Figure 3:**
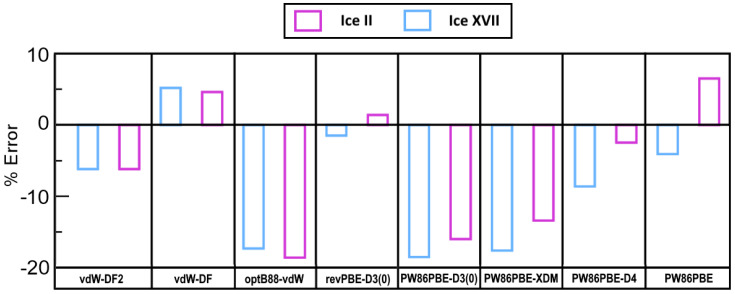
Error (in %) with respect to the computationally reported $\Delta E_{coh}\left(a_{0}^{\prime },c_{0}^{\prime }\right)$ values of −12.916/−13.7814 kcal/mol from DMC calculations for ice II/ice XVII [53].

Moving forward with the assessment, Figure 3 illustrates the percentage error concerning, as a reference, the −12.916/−13.781 kcal/mol DMC $\Delta E_{coh}\left(a_{0}^{\prime },c_{0}^{\prime }\right)$ energies for ice II/ice XVII [53]. Curiously, despite the fact that non-local functionals overestimate the lattice constant, they provide fairly accurate equilibrium energies. The functionals PW86PBE-XDM, PW86PBE-D3(0), and optB88-vdW exhibit the most significant energy underestimation, predicting values that are 2.3–2.6 kcal/mol lower than the DMC calculations. In this case, it is clearly evident that the functional which predicts the most precise energies is once again the revPBE-D3(0), being the deviation from the reference value of a mere 0.2 kcal/mol (1.4% average error).

To complete this analysis, we also discuss the B${}_{0}$ and B${}_{0}^{\prime }$ parameters, which characterize the response of a material to uniform compression, as shown in Table S1 in the Supplementary Material. They are estimated from the corresponding MEOS fits, assuming linear dependence of the bulk modulus with pressure, $B=B_{0}+B_{0}^{\prime }\times P$, with B${}_{0}$ being constant and valid for the range $B_{0}^{\prime }/2>P>0$. Thus, we observe that, with the exception of PW86PBE and PW86PBE-D4, all the functionals predict that B${}_{0}$ is greater for the He-filled structure compared to the empty one, indicating that $\mathrm{He}{}_{\left(1\right)}$@ice II is more rigid and resistant to compression than ice II, although the difference between both B${}_{0}$ values is rather small.

In turn, Lobban et al. [11] reported an isothermal bulk modulus of 14.8 GPa and 20.6 GPa for the empty and He-affected ice II structures at 200 K and 196 K, respectively. Therefore, an increase in B${}_{0}$ is expected when the ice is filled with He atoms. This can be attributed to the resistance offered by the helium atoms located within the c-axis channels against any external compression. As for the numerical values, we found that our B${}_{0}$ and B${}_{0}^{\prime }$ results are in line with previously reported zero-temperature and zero-pressure *ab initio* PW91 calculations [78], where B${}_{0}$ = 16.18 ± 0.12 GPa, with the first pressure derivative of the bulk modulus, B${}_{0}^{\prime }$, fixed equal to 6.0. Previous experimental values yielded B${}_{0}$ = 14.39 GPa for H${}_{2}$O ice II at 0.283 GPa and 237.65 K [79], B${}_{0}$ = 14.8 GPa for D${}_{2}$O ice II at 0.35 GPa and 200 K [11], and B${}_{0}$ = 14.23 GPa for D${}_{2}$O ice II at 0.35 GPa and 225 K [80]. Accordingly, the revPBE-D3(0) provides the most accurate results. On the contrary, with regards to ice XVII, there are more discrepancies. While PW86PBE, along with any dispersion scheme, and optB88-vdW DFT functionals estimate that the He-filled ice XVII is more compressible and less stiff than the empty ice XVII, the revPBE, vdW-DF and vdW-DF2 functionals suggest that B${}_{0}$ is smaller for the ice XVII, and therefore, it is less resistant to compression. Unfortunately, there is no available information in the literature regarding the response of this structure to compression. Hence, we cannot determine which of the observed trends is the expected one.

In conclusion, based on all the aforementioned information, we consider that the exchange-correlation revPBE-D3(0), which is constrained by exact functional conditions with a posteriori incorporation of the D3 dispersion corrections, is the functional that provides a better description of the interactions in both empty and singly/multiply-filled ice structures, in line with previous high-level theoretical and experimental reports on ice polymorphs [53] and interfacial water [81].

### 2.3. Influence of Increasing Number of He Atoms on Ice Crystal Properties

#### 2.3.1. Structural and Mechanical Analysis

To investigate the capacity of ice II and ice XVII structures and understand how the lattice deforms as the number of He atoms increases, different ice crystals with varying He-fillings are studied by carrying out revPBE-D3(0) calculations. In particular, in addition to the already discussed He${}_{\left(1\right)}$@ice II (1 He atom/interstice) and He${}_{\left(3/3\right)}$ice XVII (3 He atoms/interstice) systems, the He${}_{\left(2\right)}$@ice II (2 He atoms/interstice), He${}_{\left(3\right)}$@ice II (3 He atoms/interstice), He${}_{\left(1/1\right)}$@ice XVII (1 He atoms/interstice), He${}_{\left(1/2\right)}$@ice XVII (1–2 He atoms/interstice) and He${}_{\left(2/2\right)}$@ice XVII (2 He atoms/interstice) were also examined.

**Figure 4:**
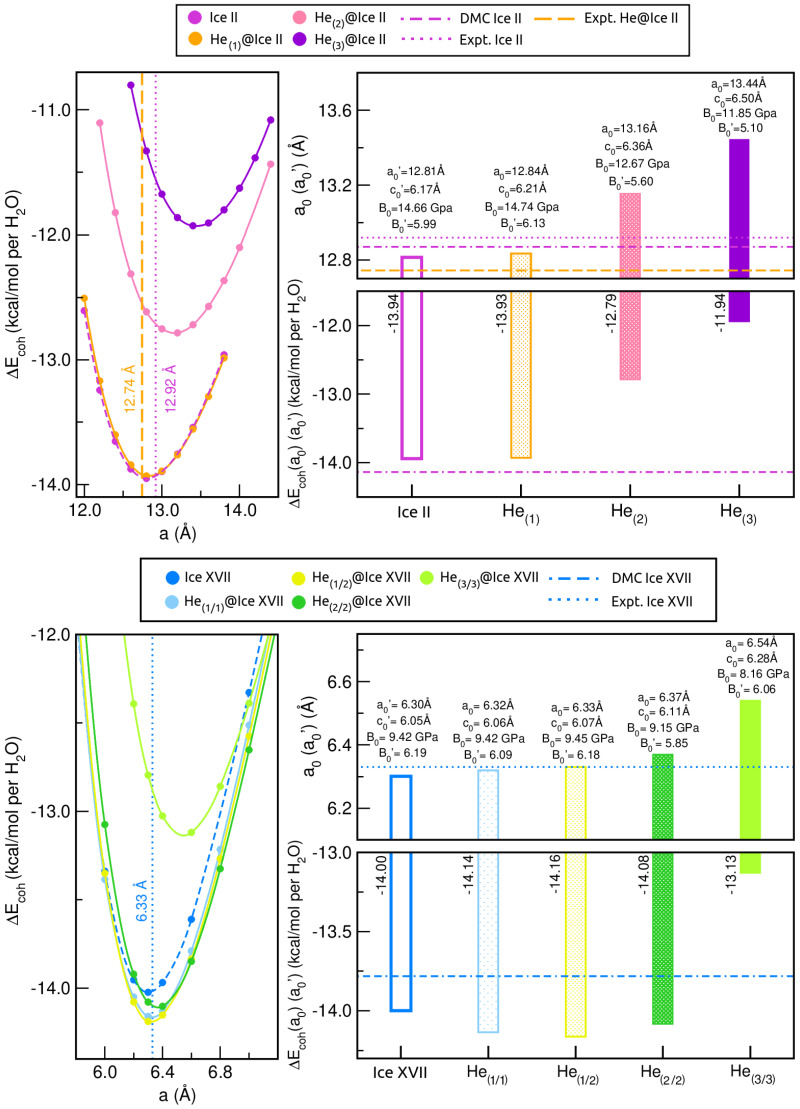
Cohesive energies (per water molecule) in kcal/mol at various values of a lattice constants for fixed values of r (r = 0.4816/0.4838 for empty/He-filled ice II and r = 0.96 for both empty/He-filled ice XVII) from revPBE-D3(0) calculations, and comparison of the resulting equilibrium a${}_{0}^{\prime }$ or a${}_{0}$ and $\Delta E_{coh}\left(a_{0}^{\prime };c_{0}^{\prime }\right)/\left(a_{0};c_{0}\right)$ parameters with the available theoretical/experimental data [2,43,53,76] for the empty and He-filled ice II (upper panels) and ice XVII (lower panels). The bulk moduli is also indicated.

**Figure 5:**
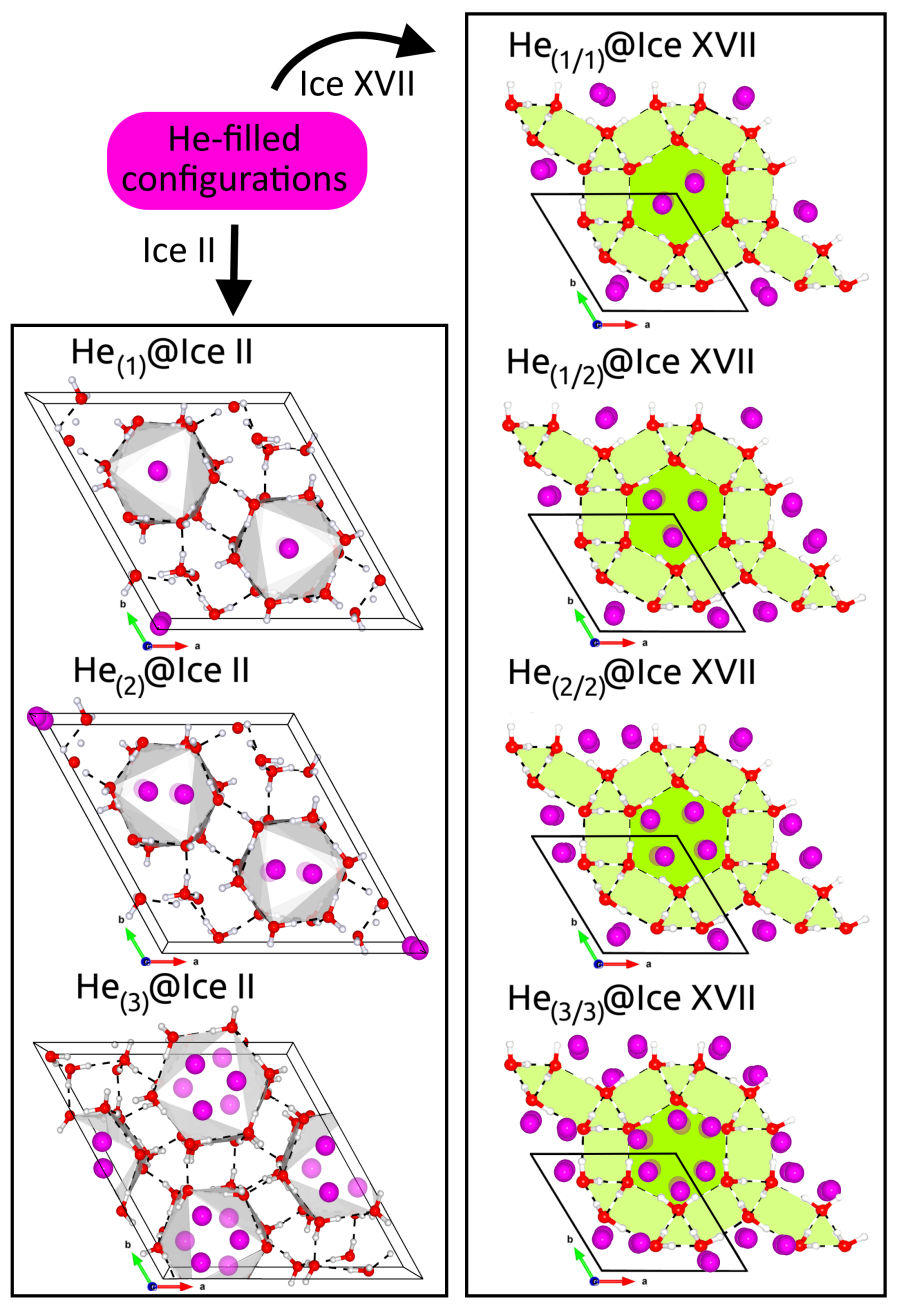
Crystalline structure of the optimized He-filled ice II (left panels) and ice XVII (right panels). Each subplot shows the unit cell of the indicated system.

Following a similar procedure to that explored in the above-mentioned benchmark study, full geometry optimisation calculations were first carried out at revPBE-D3(0) level of theory for fixed values of r (r = 0.4838/0.96 for He-filled ice II/ice XVII, respectively), within the same range of values. Subsequently, the computed optimized total energies were fitted to the MEOS, and $a_{0}$, $c_{0}$, $\Delta E_{coh}\left(a_{0};c_{0}\right)$ and B${}_{0}$/B${}_{0}^{\prime }$ were obtained. The corresponding results are graphed in Figure 4 and Figure 5, along with the findings obtained for the empty crystal in the preceding section. Numerous observations merit further discussion. When considering the ice II structure (see upper panels of Figure 4 and left-side panels of Figure 5), it is notable that the introduction of a single He atom per interstitial space barely affects the lattice; however, the addition of multiple He atoms results in significant changes to the crystal, which tends to expand in order to mitigate the repulsion generated by the He atoms in the confined space. The numerical analysis reveals that the equilibrium lattice constant for the empty ice II crystal differs from that of the He-filled He${}_{\left(1\right)}$@ice II, He${}_{\left(2\right)}$@ice II, and He${}_{\left(3\right)}$@ice II structures by 0.03, 0.35 and 0.63 Å, respectively.

The calculated lattice parameter a${}_{0}$ = 12.84 Å for He${}_{\left(1\right)}$@ice II is the most proximal to the experimentally expected value of a${}_{0}$ = 12.744(5) Å for the He-filled ice II under zero T-P conditions [2]. This value comes from the proposed expression by Londono et al. [2] to establish a relationship between lattice constant, temperature and pressure: $a_{0}=12.744\left(5\right)-0.036\left(5\right)P+0.0009\left(1\right)T$. Nonetheless, since the comparison between the expected a${}_{0}$ and a${}_{0}^{\prime }$ parameters at zero temperature and pressure is rather confusing (a${}_{0}^{\prime }$ = 12.92(2) Å according to the extrapolation made by Santra et al. [76]), it seems more reasonable to discuss about the trends observed under comparable T-P regimes [11]. Thus, alterations of 0.01, 0.02 and 0.04 Å have been reported [11] at 2.8 kbar/200 K, 4.2 kbar/250 K and 4.8 kbar/200 K between the empty and He-filled ice II crystals, respectively, leading to the expansion of the ice structure upon the incorporation of He atoms. Our results are in line with these experimental observations.

Further analysis of our equilibrium cohesive energies (per water molecule) also reveals the same conclusions. While the inclusion of one He between two hexagons along the *c*-axis has a negligible effect on the crystal, the incorporation of additional He atoms seems to be less favourable, with a difference of ∼1 and 2 kcal/mol for He${}_{\left(2\right)}$@ice II and He${}_{\left(3\right)}$@ice II, respectively, compared to the empty system. As for the bulk modulus, B${}_{0}$, filling ice II with He atoms is expected to be accompanied by an elevation in B${}_{0}$ due to the resistance exerted by the noble gas atoms under the application of external pressure [11]. This phenomenon is fulfilled when comparing the results from ice II and He${}_{\left(1\right)}$@ice II crystals; however, as additional He guests are included, a gradual decrease in B${}_{0}$ is identified, resulting in structures that are more easily deformable.

In contrast to ice II, we distinguish a less notorious He-filling effect in ice XVII, with the He${}_{\left(1/1\right)}$@ice XVII and He${}_{\left(1/2\right)}$@ice XVII lattices being only 0.02 and 0.03 Å larger than the empty one, respectively (see lower panels of Figure 4 and right-side panels of Figure 5). Incorporating 2 He atoms per interstitial space (He${}_{\left(2/2\right)}$@ice XVII) becomes more noticeable, with a size increase of 0.07 Å, whereas the introduction of three He atoms per void (He${}_{\left(3/3\right)}$@ice XVII) significantly affects the lattice by expanding it by 0.24 Å. Nonetheless, the effect is still comparatively lower than in ice II, possibly because of the wider diameter of the channels present in ice XVII, which facilitates a higher He occupancy. In the absence of any additional experimental or theoretical data for He-filled ice XVII, we can draw comparisons between our findings and those reported for Ne@ice XVII [42]. Catti et al. [42] have compared the structures of the D${}_{2}$O framework in empty and Ne-filled ice XVII, and they have reported that Ne absorption at 50 K resulted in a significant expansion of 0.05Å of c and a small contraction in the a lattice constant of approximately 0.01 Å; these changes are likely due to the Ne-D repulsion effects and the attractive van der Walls interactions between Ne and O, respectively. Here, in all the examined He@ice XVII structures, the incorporation of He atoms into the ice XVII channels results in lattice expansion, which can be attributed to the comparatively higher polarisability of Ne atoms compared to He. Such lattice expansion ensues an overall volume increase of 1.4 Å${}^{3}$ in the He${}_{\left(1/1\right)}$@ice XVII, which is in accord with that of +1.1 Å${}^{3}$ reported in partially Ne-filled deuterated ice XVII [42].

Regarding comparisons of the equilibrium cohesive energies (per water molecule) of He${}_{\left(1/1\right)}$@ice XVII, He${}_{\left(1/2\right)}$@ice XVII, and He${}_{\left(2/2\right)}$@ice XVII to the empty crystal, we found that they are more favourable by 0.14, 0.16, and 0.08 kcal/mol, respectively, while the energy difference relative to He${}_{\left(3/3\right)}$@ice XVII is 0.87 kcal/mol less energetically favoured. Therefore, while in ice II, the energies were almost identical (with only a 0.01 kcal/mol difference), the inclusion of up to 2 He atoms per interstice in ice XVII results in a more significant stabilization of the complex. Finally, we found that the bulk modulus of ice XVII and He${}_{\left(1/1\right)}$@ice XVII is equivalent, whereas for He${}_{\left(1/2\right)}$@ice XVII, it slightly increases, indicating a more rigid structure. Incorporating additional He atoms leads to more flexible systems (lower B${}_{0}$ values), which is particularly noticeable in He${}_{\left(3/3\right)}$@ice XVII. Overall, based on this analysis, it seems that He${}_{\left(3/3\right)}$@ice XVII is the least favoured structure, and has a considerable impact on the lattice in terms of its structural, energetic, and mechanical properties.

In Figures S2 and S3 of the Supplementary Material, the average H${}_{2}$O-H${}_{2}$O and He-He bond lengths for all the empty and He-filled ice II and ice XVII crystals are displayed, respectively. The ice II structure is formed of two different types of water hexagons, one with a larger average distance between opposite water molecules (flat ring), but a smaller distance between adjacent water molecules, while the other exhibits the opposite behaviour (puckered ring). Consistent with our previous analysis, we have found that there are negligible differences between the empty ice II structure and the He-filled He${}_{\left(1\right)}$@ice II system (see Figure S2). This suggests that the introduction of a single He atom per interstitial space has almost no impact on the lattice. Londono [2] and Lobban [11] have reported values for the diameter of the hexagonal rings at similar T-P conditions, finding that the He-affected flat rings were 0.04–0.05 Å larger than the unaffected ones, whereas the puckered rings remained almost unaltered. Similar observations were encountered for Ne-filled ice II lattices, where the insertion of Ne atoms resulted in an enlargement of the O-O distances and the channels diameter [82]. Our findings align with these observations, as we also predict a broadening of 0.02 Å in both rings when transitioning from the empty crystal to the He${}_{\left(1\right)}$@ice II structure. The confinement of extra He atoms significantly affects these average distances, even leading to unit cell deformation in the case of He${}_{\left(3\right)}$@ice II. Finally, an additional noteworthy feature is the positioning of the He atoms, which are situated approximately midway between the two distinct water hexagons, but with a slight displacement towards the flat ring. This aspect leads to the presence of one shorter and one longer He-He distance, in line with previous experimental findings [2].

The ice XVII structure presents wider channels, whatever the empty or He-filled system considered (see Figure S3). The average O-O bond length within water pentamers increases relative to the empty structure. Curiously, it is 0.11/0.13 Å larger for the He${}_{\left(1/1\right)}$@ice XVII/He${}_{\left(2/2\right)}$@ice XVII crystals, while only 0.01 Å in the case of He${}_{\left(1/2\right)}$@ice XVII. The He${}_{\left(3/3\right)}$@ice XVII system is much affected, with a distance increase of 0.55 Å. Equivalent to He${}_{\left(3\right)}$@ice II, it seems that the accumulation of a great number of He atoms within the small confined space of He${}_{\left(3/3\right)}$@ice XVII results in considerable repulsion and lattice distortion. The He atoms follow a spiral arrangement that conforms to the zig-zag shape of the channel. Overall, the interatomic distances found for He${}_{\left(1/2\right)}$@ice XVII are comparable to, but smaller than, those reported for Ne@ice XVII (at 50 K) [42].

**Figure 6:**
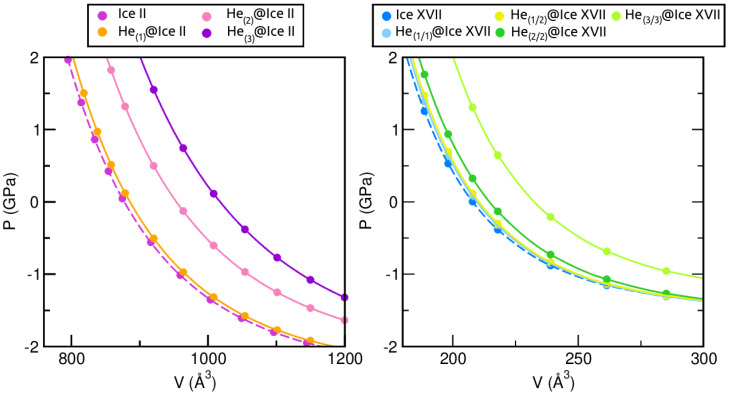
Pressure effects on the volume of the He-filled and empty ice II and ice XVII systems as determined from MEOS fits.

In turn, by using the calculated volume and bulk moduli, the pressure exerted on the ice lattices can be calculated according to the MEOS expression in terms of pressure as a function of the volume, such that $P\left(V\right)=\frac{B_{0}}{B_{0}^{\prime }}\left[{\left(\frac{V}{V_{0}}\right)}^{-B_{0}^{\prime }}-1\right]$. Figure 6 shows the volume as a function of the pressure for all the empty and He-filled ice crystals. It is well known that when a material is subjected to pressure, its volume decreases. Nevertheless, the degree of compression induced by pressure relies on the material’s properties, such as its bulk modulus. As previously mentioned, the B${}_{0}$ values encountered for ice XVII are lower than those of ice II. Consequently, it is expected that all the ice XVII structures will undergo a more significant change in volume under pressure, since they are less rigid, as confirmed in Figure 6. Besides, an increase in the number of confined He atoms results in structures that are more susceptible to compression, likely due to their reduced stability.

#### 2.3.2. Energetic Analysis

**Figure 7:**
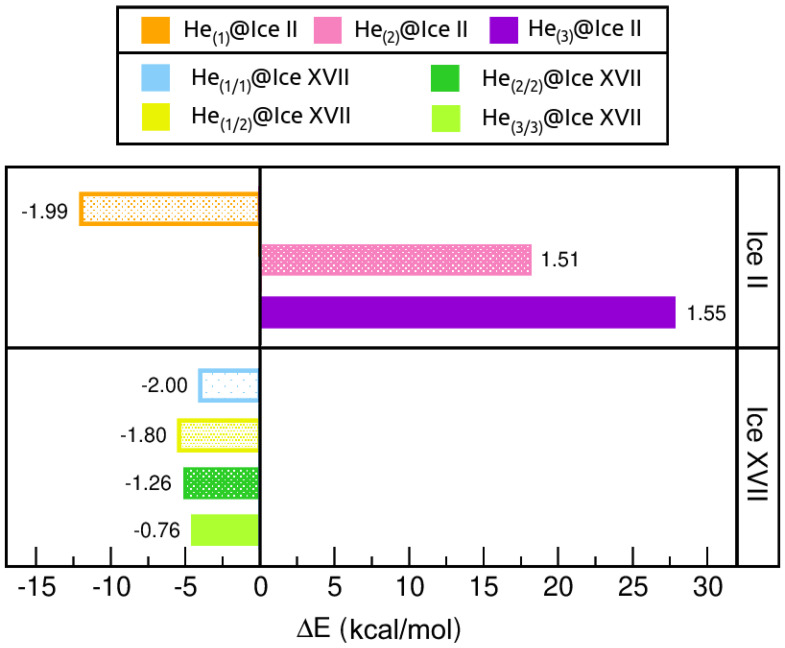
Binding energies, ΔE, in kcal/mol, for He${}_{N}$@ice II with N = 1, 2, 3 and He${}_{N}$@ice XVII with N = (1/1), (1/2), (2/2), (3/3). ΔE per He atom values are indicated inside the plot with numbers. All calculations were performed using the revPBE-D3(0) DFT functional.

The stability of these systems can be evaluated in terms of energy. To this end, the total binding energies of all the He-filled ices with respect to the guest-free systems are calculated, at the respective equilibrium lattice constants, a${}_{0}$ and c${}_{0}$, using the revPBE-D3(0) approach. The corresponding energies are shown in Figure 7. Firstly, we observe that for ice II, the only favourable He-filled structure is that containing one He atom per interstitial void, while those entrapping 2 and 3 He atoms are predicted to be energetically unstable. This observation is not surprising, since it aligns with the outcomes that have been previously discussed. On the contrary, the whole set of He-filled ice XVII configurations is energetically favoured. In particular, little difference is observed between He${}_{\left(1/2\right)}$@ice XVII, He${}_{\left(2/2\right)}$@ice XVII and He${}_{\left(3/3\right)}$@ice XVII, with He${}_{\left(1/2\right)}$@ice XVII being the optimal structure, while He${}_{\left(1/1\right)}$@ice XVII is the less favourable one. The binding energy per He atom (see numerical results in Figure 7) decreases as the number of He atoms increases; this effect is more noticeable in both He${}_{\left(2/2\right)}$@ice XVII and He${}_{\left(3/3\right)}$@ice XVII structures.

**Figure 8:**
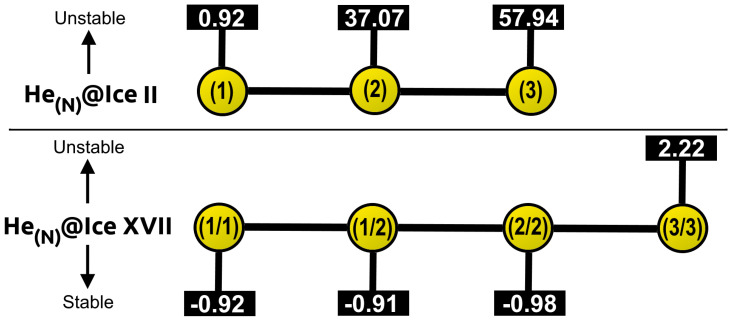
Saturation energies, $\Delta E_{sat}$, in kcal/mol, for He${}_{N}$@ice II with N = 1, 2, 3 and He${}_{N}$@ice XVII with N = (1/1), (1/2), (2/2), (3/3), as computed from the present revPBE-D3(0) calculations.

Further analysis of the stability entails the computation of the saturation energies for all He-filled structures, as illustrated in Figure 8. Such values provide an indication of the overall energy gain upon saturation of all channels, rather than just with respect to the addition of individual He atoms. In contrast to the binding energies, unfavourable $\Delta E_{sat}$ is predicted for all He${}_{N}$@ice II systems. However, its value is in close proximity to zero for the He${}_{\left(1\right)}$@ice II, while it experiences a sharp increase for He${}_{\left(2\right)}$@ice II and He${}_{\left(3\right)}$@ice II, reaching values that indicate extreme instability. The He${}_{N}$@ice XVII ensembles remain stable for N = (1/1), (1/2) and (2/2), presenting energies of similar magnitudes. Among them, He${}_{\left(2/2\right)}$@ice XVII exhibits the most favourable configuration. Conversely, the He${}_{\left(3/3\right)}$@ice XVII structure is estimated to be unstable, with a $\Delta E_{sat}$ value of 2.22 kcal/mol.

In general, considering the energetic and structural evaluation of both empty and He-filled ice II and ice XVII crystals, it seems that the ice II only may allow the inclusion of one He atom per interstitial space, while the ice XVII could accommodate up to two He atoms per void. It is important to consider that the present findings are applicable only under zero temperature and pressure conditions. According to the phase diagram of water, the ice XVII structure is situated in close proximity to these conditions. Hence, it is understandable that our calculations predict that the formation of He-filled ice II, which occurs under extremely high-pressure conditions, is less favourable.

## 3. Summary and Conclusions

This research focused on examining the behavior of crystalline ice structures confining an increasing number of He atoms. We have obtained valuable first-principles insights into the stability of two specific systems: the previously synthesized He@ice II, and a recent hypothetical He@ice XVII structure. First of all, we have conducted a benchmark study, including various semi-local dispersion-corrected GGA and non-local van der Waals-inclusive DFT-D functional approaches, in order to identify the most appropriate one that adequately describes the hydrogen bonds and van der Waals interactions inherent in these systems. Our findings indicate that the revPBE-D3(0) approach yields remarkably accurate results in comparison with available theoretical and/or experimental data. Benchmark studies on the performance of different DFT approaches can assist in resolving controversial issues and/or guide the development of novel/improved functionals.

In turn, the stability of different configurations of ice II and ice XVII structures with varying numbers of trapped He atoms within their channels was evaluated. The analysis primarily focused on examining structural, mechanical, and energetic properties, such as equilibrium lattice constants, resistance to compression, and binding/saturation energies. The presence of He atoms within the interstitial spaces of these systems has been observed to have a noteworthy impact. On the one hand, the ice II structure only accepts the inclusion of only one He atom per void, leading to minimal stabilization of the structure. The introduction of additional He atoms results in highly unstable configurations, causing even deformation of the lattice structure. On the other hand, the larger diameter of ice XVII allows for inclusion of up to two He atoms per interstitial space. However, the confinement of extra guest atoms produces a destabilizing effect. Moreover, in contrast to ice II, the addition of just one He atom exerts a significant stabilizing effect on the lattice.

Overall, while both ice II and ice XVII structures are predicted to exhibit stability in the presence of He atoms, ice XVII demonstrates more stable configurations under zero temperature and pressure conditions. This observation aligns with the expected behavior in the phase diagram of solid water.

To further advance this line of investigation, additional studies can focus on several aspects. Vibrational properties can be explored by conducting phonon calculations to analyze the behavior of lattice vibrations within the He-confined ice structures. Furthermore, examining the response of these systems to variations in temperature and pressure would provide valuable insights into their thermodynamic stability and phase transitions. Molecular dynamics simulations can be employed to investigate the diffusion behavior of helium atoms within the channels, shedding light on their mobility and migration pathways. Understanding the stabilising role of He atoms in these solid hydrostructures holds great potential for the discovery of new low-density ices and materials.

## Data Availability

The data supporting reported results are available from the corresponding author upon request. Cohesive energies and parameters obtained from the MEOS fitting to semi-local and non-local DFT functionals for both both He-filled and empty ice II and ice XVII are available online at https://www.mdpi.com/article/10.3390/molecules28237893/s1.

## Supplementary Materials

The following supporting information can be downloaded at: https://www.mdpi.com/article/10.3390/molecules28237893/s1. Figure S1: Cohesive energies of the He-filled (left panels) and empty (right panels) ice II and ice XVII as a function of the lattice constant a and ratio r. Symbols indicate the computed values from the PW86PBE/-D4/-XDM calculations, Figure S2: Average bond length and distances in Å for the empty and He-filled ice II structures considered in this study, Figure S3: Average bond length and distances in Å for the empty and He-filled ice XVII structures considered in this study, Table S1: MEOS fitting results obtained from semi-local and non-local DFT functionals for both He-filled and empty ice II and ice XVII.
