# Supplementary material for: Confining He Atoms in Diverse Ice-Phases: Examining the Stability of He Hydrate Crystals through DFT Approaches
Source: Molecules. 2023 Dec 1;28(23):7893. doi: 10.3390/molecules28237893 (PMC10708247; doi:10.3390/molecules28237893)
Supplement: Supplementary file 1 [file molecules-28-07893-s001.zip › molecules-2727065-supplementary.pdf]

Confining He Atoms in Diverse Ice-phases: Examining the Stability of He Hydrate Crystals Through DFT Approaches

Raquel Yanes-Rodríguez[1 a] and Rita Prosmiti[1*]

[1] *Institute of Fundamental Physics (IFF-CSIC), CSIC, Serrano 123, 28006 Madrid, Spain*

E-mail: *rita@iff.csic.es;Phone:+34-91-5616800Ext.442292

[a] Doctoral Programme in Theoretical Chemistry and Computational Modelling, Doctoral School, Universidad Autnoma de Madrid

SUPPLEMENTARY MATERIAL

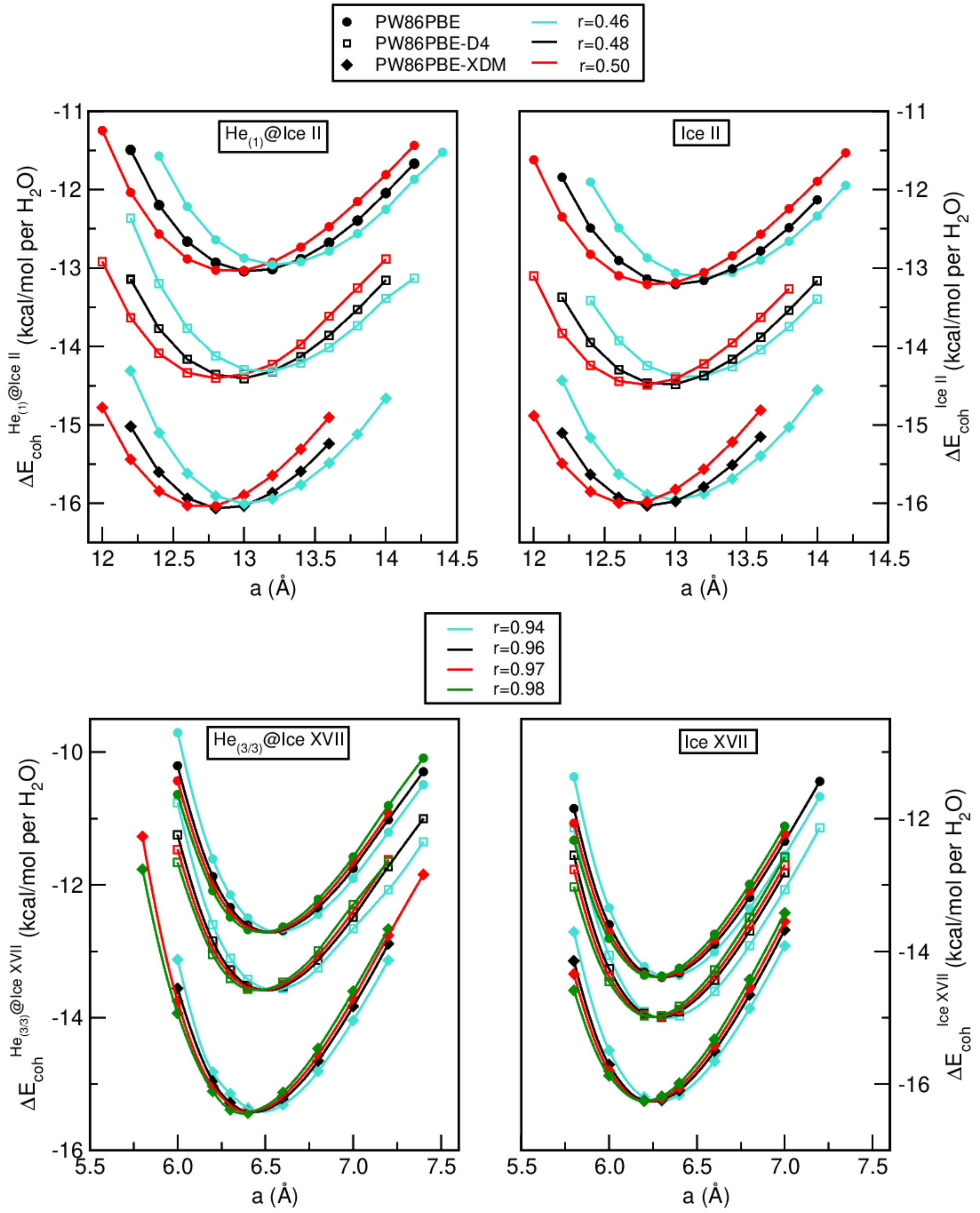

Figure S1: Cohesive energies of the He-filled (left panels) and empty (right panels) ice II and ice XVII as a function of the lattice constant a and ratio r. Symbols indicate the computed values from the PW86PBE/-D4/-XDM calculations.

Table S1: MEOS fitting results obtained from semi-local and non-local DFT functionals for both He-filled and empty ice II and ice XVII.

System	Parameter	PW86PBE	PW86PBE-D4	PW86PBE-XDM	PW86PBE-D3(0)	revPBE-D3(0)	optB88-vdW	vdW-DF	vdW-DF2
**Ice II**	a_0' / c_0' (Å)	13.00/6.26	12.90/6.21	12.81/6.17	12.77/6.15	12.81/6.17	12.66/6.10	13.32/6.42	13.12/6.32
	$\Delta E_{coh}(a_0', c_0')$ (kcal/mol)	-13.21	-14.49	-16.03	-16.40	-13.94	-16.76	-13.49	-15.01
	B_0 (GPa)	15.47	16.57	19.48	19.70	14.66	19.45	12.16	15.94
	B_0'	5.71	5.94	5.57	5.35	5.99	5.70	4.85	5.03
$He_{(1)}$**@ice II**	a_0/c_0 (Å)	13.03/6.30	12.92/6.25	12.82/6.20	12.76/6.17	12.84/6.21	12.67/6.13	13.34/6.45	13.12/6.35
	$\Delta E_{coh}(a_0, c_0)$ (kcal/mol)	-13.05	-14.41	-16.07	-16.53	-13.93	-17.02	-13.85	-15.24
	B_0 (GPa)	15.15	16.56	19.66	20.03	14.74	19.76	12.35	16.32
	B_0'	5.71	5.98	5.73	5.81	6.13	5.77	4.93	5.43
**Ice XVII**	a_0'/c_0' (Å)	6.29/6.04	6.28/6.03	6.24/5.99	6.23/5.98	6.30/6.05	6.18/5.93	6.47/6.21	6.42/6.17
	$\Delta E_{coh}(a_0', c_0')$ (kcal/mol)	-14.36	-14.98	-16.22	-16.34	-14.00	-16.18	-13.07	-14.64
	B_0 (GPa)	11.29	11.79	12.92	12.82	9.42	11.27	7.00	8.75
	B_0'	6.28	5.89	6.03	6.08	6.19	5.11	4.16	3.67
$He_{(3/3)}$**@ice XVII**	a_0/c_0 (Å)	6.75/6.48	6.72/6.52	6.66/6.39	6.61/6.34	6.54/6.28	6.59/6.33	6.93/6.65	6.78/6.51
	$\Delta E_{coh}(a_0, c_0)$ (kcal/mol)	-12.67	-13.54	-15.41	-16.01	-13.13	-16.04	-13.99	-15.04
	B_0 (GPa)	8.43	8.86	10.89	11.65	8.16	10.15	7.25	9.37
	B_0'	6.39	6.69	5.71	6.62	6.06	5.89	5.61	5.73

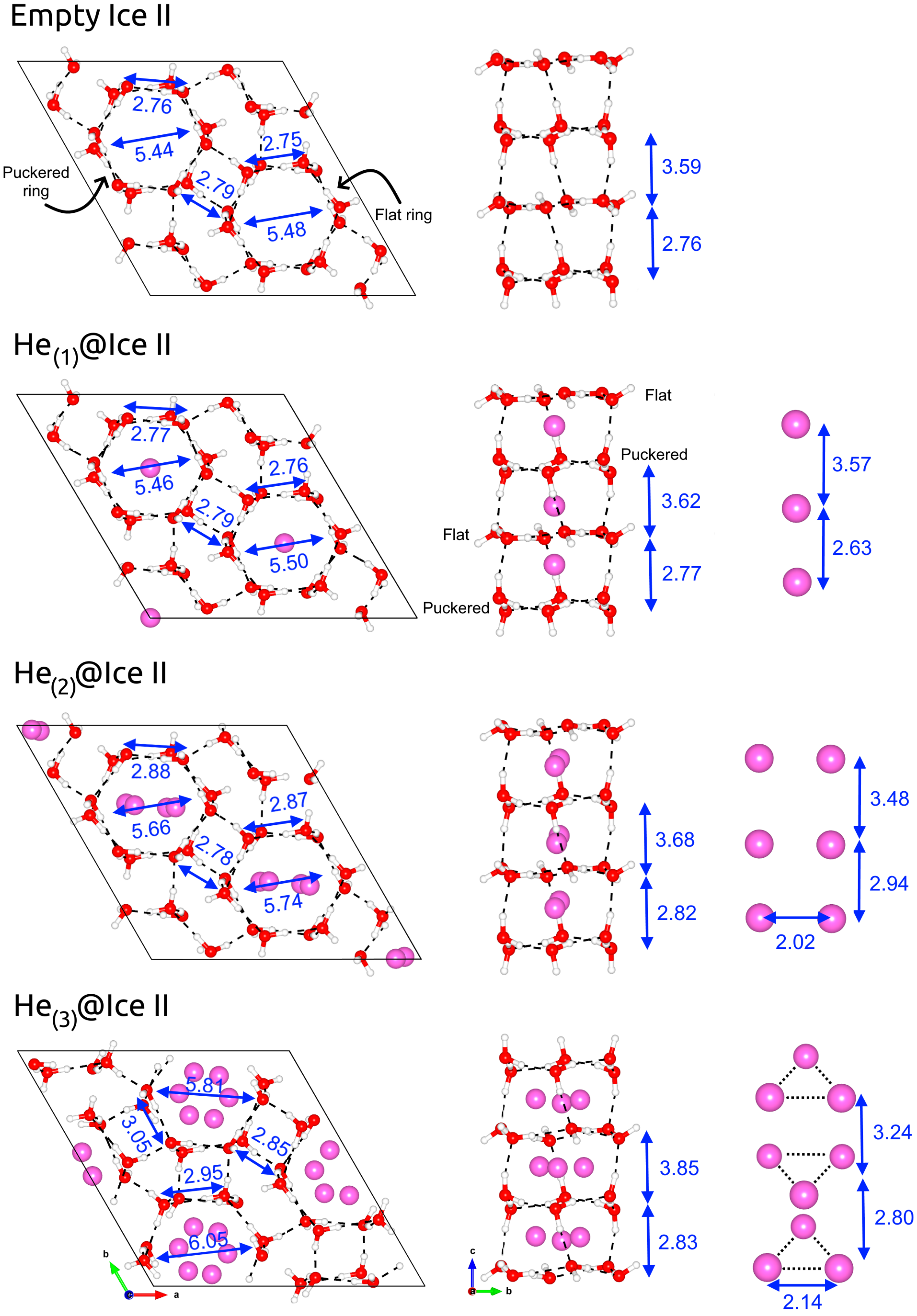

Figure S2: Average bond length and distances in Å for the empty and He-filled ice II structures considered in this study.

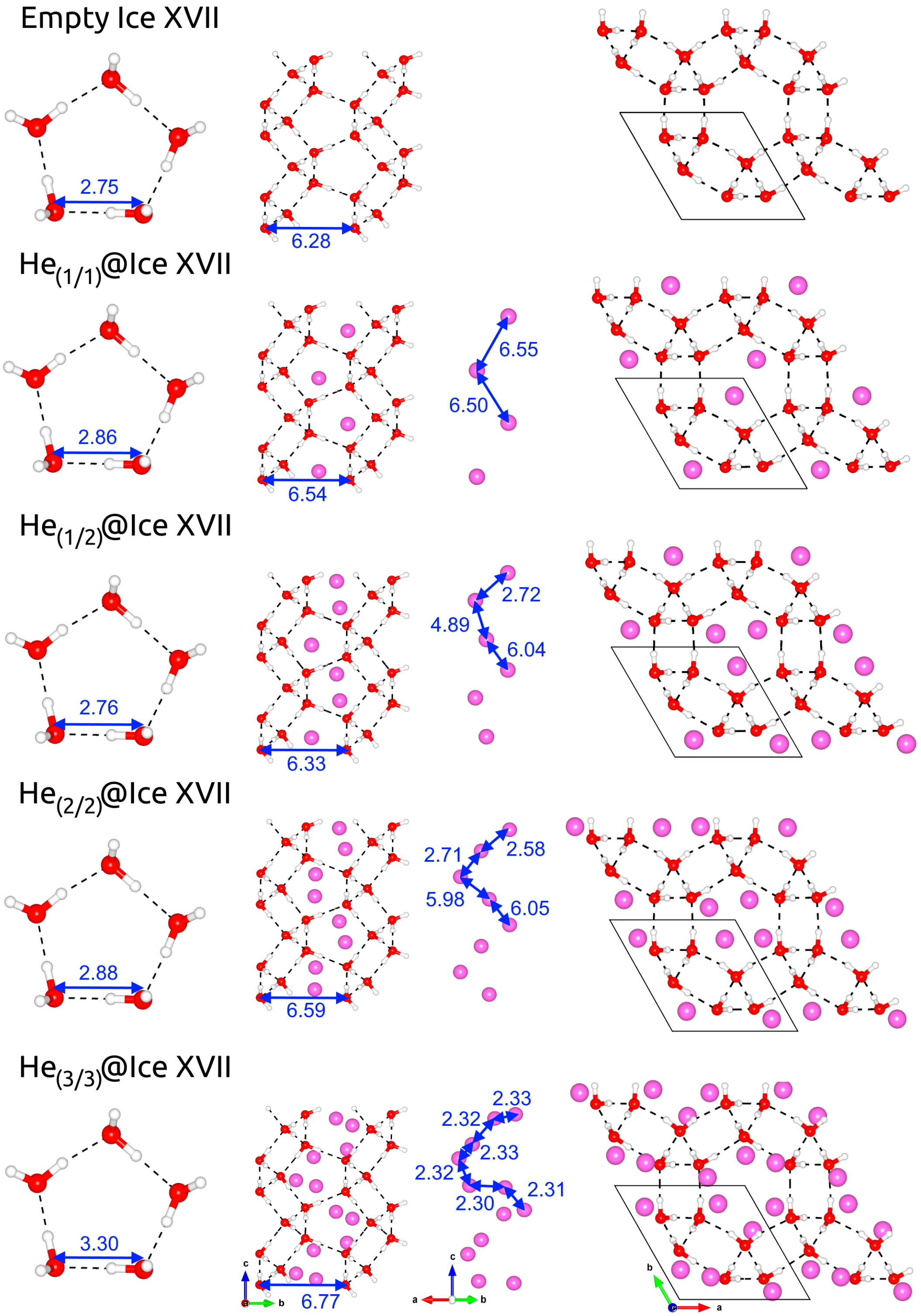

Figure S3: Average bond length and distances in Å for the empty and He-filled ice XVII structures considered in this study.
